# Differential Gene Expression by RamA in Ciprofloxacin-Resistant *Salmonella* Typhimurium

**DOI:** 10.1371/journal.pone.0022161

**Published:** 2011-07-19

**Authors:** Jie Zheng, Fei Tian, Shenghui Cui, Jiuzhou Song, Shaohua Zhao, Eric W. Brown, Jianghong Meng

**Affiliations:** 1 Joint Institute for Food Safety and Applied Nutrition, and Department of Nutrition and Food Science, University of Maryland, College Park, Maryland, United States of America; 2 Department of Animal and Avian Science, University of Maryland, College Park, Maryland, United States of America; 3 State Food and Drug Administration, Beijing, China; 4 Center for Veterinary Medicine, U.S. Food and Drug Administration, Maryland, University of Maryland, College Park, Maryland, United States of America; 5 Center for Food Safety & Applied Nutrition, U.S. Food & Drug Administration, Maryland, University of Maryland, College Park, Maryland, United States of America; Monash University, Australia

## Abstract

Overexpression of *ramA* has been implicated in resistance to multiple drugs in several enterobacterial pathogens. In the present study, *Salmonella* Typhimurium strain LTL with constitutive expression of *ramA* was compared to its *ramA*-deletion mutant by employing both DNA microarrays and phenotype microarrays (PM). The mutant strain with the disruption of *ramA* showed differential expression of at least 33 genes involved in 11 functional groups. The study confirmed at the transcriptional level that the constitutive expression of *ramA* was directly associated with increased expression of multidrug efflux pump AcrAB-TolC and decreased expression of porin protein OmpF, thereby conferring multiple drug resistance phenotype. Compared to the parent strain constitutively expressing *ramA*, the *ramA* mutant had increased susceptibility to over 70 antimicrobials and toxic compounds. The PM analysis also uncovered that the *ramA* mutant was better in utilization of 10 carbon sources and 5 phosphorus sources. This study suggested that the constitutive expression of *ramA* locus regulate not only multidrug efflux pump and accessory genes but also genes involved in carbon metabolic pathways.

## Introduction

Active efflux by AcrAB-TolC plays an important role in conferring multidrug resistance (MDR) in salmonellae [1]. The AcrAB-TolC efflux system also contributes to *Salmonella* pathogenesis [2]. Mutants lacking *acrA*, *acrB*, or *tolC* were attenuated via differential expression of major operons and proteins involved in pathogenesis globally [3].

To date, our knowledge on the regulation of expression of AcrAB has mostly come from work carried out in *Escherichia coli* [4], [5], [6]. In addition to *marA* and *soxS* [7], *ramA*, a species-restricted and encoding a 113-amino-acid regulatory protein [8], has been implicated in MDR in *Salmonella* and other bacteria [7], [9], [10], [11], [12]. Increased *ramA* expression has been noted among clinical isolates of *Klebsiella pneumoniae* [13], [14] and *S*. Typhimurium [15]. The *ramA* locus, when cloned in *E. coli*, elicited resistance to multiple antibiotics, decreased expression of the OmpF porin, and increased expression of the efflux AcrAB [16]. Ricci et al. showed that RamA was required to select MDR mutants after exposure to substrates of the AcrAB-TolC pump [17]. However, van der Straaten et al. reported that inactivation of *ramA* in wild-type *S*. Typhimurium failed to confer increased susceptibilities to antibiotics [11]. Recently, it has been shown that mutations in *ramR*, a tetR-like repressor of *ramA* [18], [19] and mutations in *ramA* promoter region [18], [20] resulted in overexpression of *ramA*, thereby conferring MDR phenotype through induction of *acrAB* and *tolC*. Additionally, van der Straaten et al. [21] showed that *soxR/S* was not the only regulon involved in protective response of *Salmonella* to macrophage-derived oxidative stress and that *ramA* played an important role in resistance to superoxide and nitrogen intermediates produced by phagocytes as well.

Barbosa and Levy [22] reported differential expression of over 60 chromosomal genes in the MarA regulon of *E. coli*. The *mar* and *soxRS* systems exert overlapping effects on the regulation of efflux pumps and porin syntheses in *E. coli* [23]. While in *S*. Typhimurium, Nikaido et al. demonstrated that induction of *acrAB* by indole was regulated by *ramA*, independent of *marA*, *soxS*, or *rob* [24]. Furthermore, Bailey et al. [25] recently showed that following disruption of *ramR*, or artificial overexpression of *ramA* in *S*. Typhimurium, global changes in expression of genes involved in MDR efflux, virulence, and amino acid biosynthesis were observed [26]. However, the relationships between *ramA* and *marA,* and *ramA* and *soxRS* remain unclear in *Salmonella*.

The goal of this study was to examine both global genotypic and phenotypic changes associated with inactivation of *ramA*. Data interrogation of DNA microarray and Biolog Phenotype MicroArray after growth of *S*. Typhimurium LTL and a derivative lacking *ramA* revealed that constitutive expression of *ramA* affected expression of genes not only involved in multidrug resistance, but genes associated with carbon and phosphorus metabolism.

## Materials and Methods

### Bacterial strains

S. Typhimurium strain LT2 was whole-genome sequenced and kindly given by The Institute for Genomic Research (now The J. Craig Venter Institute, JCVI). LTL (ciprofloxacin MIC: 4 µg/ml) with a single point mutation in GyrA was derived from S. Typhimurium LT2 by in vitro-selection using ciprofloxacin [20]. Strain LTL represented the control, expressing RamA constitutively. Experimental strain LTL*ramA::aph* was constructed using λ red site-specific recombination, as previously described [27], in which the *ramA* gene was replaced with an *aph* cassette expressing kanamycin resistance. Replacement of target gene, *ramA*, was verified by PCR using the k1 and k2 primers and primers flanking the deleted regions [20].

### RNA isolation and mRNA purification

Overnight bacterial cultures were diluted 1∶100 in fresh Difco Mueller-Hinton broth (Becton Dickinson and Co., Sparks, MD) and grown to mid-logarithmic phase (A_600_ = 0.4–0.45) at 37°C. Cells were harvested using an RNAprotect reagent (Qiagen, Valencia, CA) and processed for microarray real-time PCR analyses. Total RNA was isolated using TRIZOL (Invitrogen, Carlsbad, CA) and cleaned using the RNeasy mid-kit (Qiagen) according to the manufacturers' instructions. RNA preparations were treated with RNase-free DNase (Qiagen) on columns to remove genomic DNA (gDNA) contamination. Additional PCR reaction was conducted to confirm the loss of gDNA. RNA samples were quantified using an ND1000 spectrophotometer (NanoDrop Technologies, Wilmington, DE) and further inspected by gel electrophoresis. For microarray study, ribosomal RNA (rRNA) was extracted from total RNA to increase the sensitivity of the DNA microarray using the MicrobExpress bacterial mRNA purification kit (Ambion, Austin, TX). The quality and quantity of the mRNA samples were assessed using gel electrophoresis.

### cDNA synthesis, dye labeling and array hybridization

Conversion of mRNA to cDNA and cDNA labeling was performed according to a publicly available microbial microarray protocol (http://pfgrc.jcvi.org/index.php/microarray/protocols.html). Briefly, 200 ng of purified mRNA was converted into cDNA in a total volume of 30.1 µl using SuperScript™ III first-strand synthesis system (Invitrogen), random hexamer primers, and dNTP/aa-UTP labeling mix (Sigma, St. Louis, MO). Amplified aminoallyl-labelled cDNA was then coupled to dye Cy3 or Cy5. Only cDNA samples with >800 pmol of dye incorporation, and<20 in number of nucleotides/dye incorporation ratio (pmol cDNA/pmol Cy dye) was used in hybridization. The Cy3/Cy5 probe mixture was then dried in a speed vac and resuspended in a hybridization buffer. *S.* Typhimurium/Typhi slides [28] (v.5.0) were obtained from the Pathogen Functional Genomics Resource Center (PFGRC) at the J. Craig Venter Institute. Each array slide consists of 5462 70-mer oligonucleotide representing 99% of the open reading frames of the *S*. Typhimurium LT2 genome. (http://pfgrc.jcvi.org/index.php/microarray/array_description/salmonella_typhimurium/version5.html). The labeled cDNAs were hybridized to arrays according to a protocol developed at the Institute (http://pfgrc.jcvi.org/index.php/microarray/protocols.html). Briefly, slides were pre-hybridized in pre-warmed prehybridization buffer (5×SSC buffer, containing 1% BSA and 0.1% SDS) for 1 h at 42°C, followed by washing with MilliQ/DI water and drying with centrifugation. Hybridization was performed in a hybridization chamber (Corning, Pittsburgh, PA) at 42°C for 16–20 h using a hybridization buffer containing 5×SSC, 0.1% SDS, 25% (v/v) formamide, 0.1 mM DTT and 0.6 µg/µl sheared salmon sperm DNA. The slides were then washed, and spun dry. The experiment was repeated six times (e.g., biological replication) with duplicates in each time for a dye-swap technical replication.

### Image acquisition and data analysis

Microarrays were scanned with two wavelengths for Cy3 (570 nm) and Cy5 (660 nm) using a laser fluorescent ScanArray 4000 scanner (GSI Lumonics, Novi, MI). Average signal intensity and local background measurements were obtained for each spot on the array using Spotfinder software (www.tm4.org/software/) to generate.mev files from TIFF array images. Analyses were performed using Limma (www.bioconductor.org) [29]. The LOWESS method was employed to correct intensity-dependent color dye effects for the remaining arrays [30]. Between-array normalization was conducted with quartile method [31]. Moderated t-statistics were calculated with an empirical Bayes method [29] to alleviate the bias due to the variance with low intensities. Student's *t*-test was used to derive *P* values, which were adjusted for false discovery rate (FDR) at 5% [32]. Only genes whose ratios were ≥2-fold changes (either increased or decreased) with 99% confidence (P<0.01) were considered statistically significant. Array data were deposited at the GEO database of the National Center for Biotechnology (http://www.ncbi.nlm.nih.gov/geo) under the series number GSE15485.

### Quantitative real-time PCR

A total of 22 genes were randomly selected for real-time PCR analysis to validate microarray results using a previously described method [33]. Real-time PCR was also used to confirm the relationship of small RNA gene *micF* with *ramA* since *micF* was not spotted on the slides. Following a reverse transcription reaction of total RNA, resultant cDNAs were diluted 10^3^-fold, except those of the housekeeping gene (*rrsG*, a 16S rRNA gene) which underwent a 10^6^-fold dilution. Real-time PCR was performed using the IQ5 multicolor real-time PCR system (Bio-Rad, Hercules, CA) with each of the specific primer pairs described in Table S1, 5 µl of cDNA, and iQ SYBR Green Supermix (Bio-Rad). PCR conditions included 3 min at 95°C, followed by 40 cycles of 95°C for 10 s, 50°C, 58°C, or 60°C for 15 s, and 72°C for 30 s. Transcription levels of target genes were normalized using *rrsG* as an internal standard [7], [34]. Efficiency of amplification was determined for each primer set, and melting curve was conducted to confirm the absence of primer-dimer formation. The ΔΔC_t_ method was used to calculate fold induction of transcription of a target gene by comparison to a value relative to the parent strain grown in MH broth at log-phase. Correlation coefficient was determined by comparison of real-time PCR results with microarray data.

### Phenotyic microarray analysis

LTL and LTL*ramA*::*aph* were examined for cellular phenotypes using Biolog Phenotypic Microarray (PM) System (Biolog, Inc., Hayward, CA), which allows for the simultaneous screening of approximately 1,200 phenotypes [35]. All materials, media, and reagents for the PM system were purchased from Biolog. PM experiments were conducted using conditions recommended by the manufacturer [36]. The PM plates were incubated at 37°C in an Omnilog incubator and readings were recorded every 15 min for 48 h. Bacterial respiration was assessed within each well by monitoring color formation resulting from reduction of the tetrazolium violet (dye A), and color intensity was expressed in arbitrary units (AU). Kinetic data were analyzed with OmniLog-PM software from Biolog (OL_PM_Par1.20.02, Dec. 08, 2005). Based upon work by Zhou et al. [36] and Bailey et al.[25], substrates showing ±1.5-fold (≥15000 area under curve, arbitrary units) difference between LTL and *ramA* mutant were considered as a different phenotype. The tests were repeated once to confirm phenotypes detected with the metabolic arrays (PM1 to PM8).

Bioscreen C (Growth Curves USA, NJ) was performed using chemically defined M9 minimal medium to determine utilization of selected carbon and phosphorus sources. To confirm the mutant phenotypes detected on the inhibitor sensitivity arrays (PM9 to PM20), 1.33-fold serial dilutions of selected chemicals were used in 96-well microplates. Agar dilution was also used to test susceptibility to several antimicrobial compounds on MH agar [37].

## Results

### Effects of *ramA* deletion on the transcriptome

The microarray data revealed that approximately 0.7% of the genes in the *S*. Typhimurium LTL genome displayed ≥2-fold differential expression with a P value<0.01 when *ramA* was inactivated by inserting kanamycin resistance gene, *aph* (Table 1). Notably, the number of genes with decreased expression (n = 21) were more than that of genes with increased expression (n = 12) by *ramA* knockout.

**Table 1:** Gene expression variations greater than or equal to 2-fold due to *ramA* inactivation in comparing *S*. Typhimurium strain LTL*ramA::aph* to *S*. Typhimurium strain LTL.

Locus tag in*S*. Typhimurium	Annotation	Adjusted P-value	Gene name	Fold change
STM0581	putative regulatory protein	3.09E-16	*ramA*	−4.8
STM3179^a^	NADPH specific quinone oxidoreductase (drug modulator)	4.25E-16	*mdaB*	−3.2
STM0874^a^	oxygen-insensitive NADPH nitroreductase	1.24E-18	*mdaA/nfsA*	−3.2
STM0476a, b	acridine efflux pump	8.42E-14	*acrA*	-3.2
STM0475a, b	RND family, acridine efflux pump	1.98E-15	*acrB*	−3.1
STM3276^a^	putative alkanal monooxygenase	1.54E-12	*yhbW*	-3.0
STM0873	putative inner membrane protein	2.95E-13	*ybjC*	−2.9
STM3186a, b	outer membrane channel specific tolerance to colicin	7.89E-15	*tolC*	-2.8
STM3313	putative ABC superfamily (atp_bind) transport protein	1.58E-15	*yrbF*	−2.5
STM0156	putative outer membrane protein	5.03E-13		-2.5
STM3312	putative ABC superfamily (membr) transport protein	2.21E-14	*yrbE*	−2.5
STM0509	putative outer membrane protein (porin)	1.64E-15		-2.4
STM3180	putative cytoplasmic protein	7.83E-13	*ygiN*	−2.3
STM0215^a^	methionine aminopeptidase	1.15E-13	*map*	-2.3
STM0780	putative outer membrane or exported	3.61E-13		−2.2
STM0492	putative CPA2 family transport protein	3.17E-13	*ybaL*	-2.2
STM3311	putative ABC superfamily (bind_prot) transport protein	2.16E-15	*yrbD*	−2.1
STM1004	nicotinate phosphoribosyltransferase	4.43E-11	*pncB*	-2.1
STM1651^b^	putative pyruvate-flavodoxin oxidoreductase	5.61E-13	*nifJ*	−2.0
STM2203^a^	endonuclease IV	2.11E-14	*nfo*	-2.0
STM0875	ribosomal protein S6 modification protein	1.15E-13	*rimK*	−2.0
STM4231^a^	phage lambda receptor protein maltose high-affinity receptor	5.98E-07	*lamB*	2.0
STM4330	chaperone Hsp60 with peptide-dependent ATPase activity	1.54E-08	*groEL*	2.0
STM4329	chaperone Hsp10, affects cell division	2.76E-09	*groES*	2.1
STM0164	putative transcriptional regulator (LysR family)	7.34E-11		2.1
STM0162	putative inner membrane protein	1.57E-09		2.2
STM2665	ribosome associated factor	9.95E-06	*yfiA*	2.3
STM0974	putative FNT family, formate transporter	8.61E-08	*focA*	2.4
STM0543	major type 1 subunit fimbrin (pilin)	7.97E-15	*fimA*	2.4
STM2261	ferredoxin-type protein: electron transfer	1.26E-06	*napF*	2.5
STM0163	4-hydroxythreonine-4-phosphate dehydrogenase	1.40E-10	*pdxA*	2.5
STM0999^a^	outer membrane protein F precursor	1.19E-14	*ompF*	3.2
STM2646	putative formate acetyltransferase	8.51E-07	*yfiD*	3.6

a genes co-regulated by *marA* and *soxS* in *E. coli*.

b genes also regulated by soxS in *S*. *enterica* serotype Typhimurium.

Genes affected by the *ramA* inactivation were dispersed throughout the genome (Table 1). The differentially expressed genes with known COG function were categorized into 11 functional groups, mainly including inorganic ion, coenzyme, and carbohydrate transport and metabolism, energy production and conversion, cell wall/membrane biogenesis, multifunctional, secondary metabolites biosynthesis, transport and catabolism.

**Figure 1:**
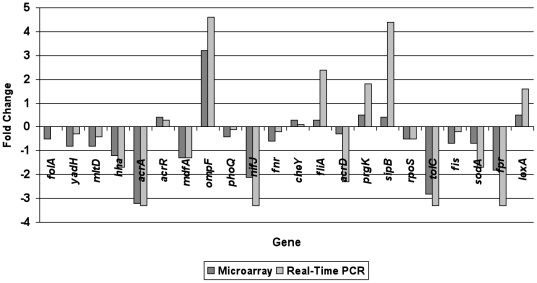
Comparison in expression ratios of 22 randomly selected genes between microarray and real time-PCR. The array fold-changes (in dark grey box) were based on averages from six biological replications. The fold differences (ΔC_T_) in expression levels of the genes tested using real time-PCR (in light grey box) were calculated from triplicate reactions against C_T_ value of housekeeping gene (*rrsG*) of *S*. Typhimurium. *acrA*, *tolC* and *ompF* are genes showing significant difference (≥2-fold with P≤0.01) in microarray data.

Real-time quantitative RT-PCR was used to corroborate selected values from microarrays. The correlation in the expression ratios of 22 genes between the microarray and real time-PCR was measured using Student's *t*-test. The results showed that there was a high degree of concordance (*r* = 0.883) between data from the two methodologies (γ = 0.87, *P*<0.05) (Fig. 1).

### Changes in the transcript abundance of genes regulated among MarA, SoxS, and RamA

*marA*, *soxS,* and *ramA* are transcriptional activators from the family of AraC/XylS. There are over 60 known genes under *marA* regulation and 15 under *soxS* regulation in *E. coli* [22], [38], [39]. Many oxidative stress genes responding to SoxS are also reactive to MarA in *E. coli*. Our data revealed that at least 10 genes with diverse physiological functions regulated directly or indirectly by both the MarA and SoxS regulators in *E. coli* [22], [40], [41] were also affected by RamA in *S*. Typhimurium (Table 1). In particular, the transcription level of MDR related genes *acrA*, *acrB* [42], and *tolC* [42], [43] decreased; while *ompF,* encoding an outer membrane porin protein, was activated in the *ramA* mutant. Real-time PCR showed 2.3±0.1-fold down-regulation of *micF* transcription in the *ramA* mutant, which confirmed the role of small RNA encoding gene *micF* in the regulation of OmpF reported in *E. coli* [22], [44], [45]. No significant change was observed in the expression of *acrR*, encoding a local repressor of AcrAB [46]. Furthermore, the transcription level of *nifJ*, a putative oxidoreductase, regulated by SoxS only in *S.* Typhimurium [47], decreased by 2-fold in the *ramA::aph* mutant (Table 1). While the abundance of genes that encode proteins important to stress response (e.g., *groEL*, *groES*) (Table 1) and are known to be induced by the presence of SylA in *S*. Typhimurium [48], were significantly higher in the *ramA::aph* mutant than in the parent strain with over-expression of *ramA*. And anaerobic metabolism related gene *napF* (Table 1) was also positively affected by the *ramA* knockout, which is consistent with the effect of *acrA* knockout described previously [3].

### Phenotypic characterization of *ramA* mutant

Of the 1,200 phenotypes screened, 114 phenotypes showed ≥1.5-fold difference in tetrazolium dye reduction in LTL*ramA*::*aph* compared to parental strain LTL. Resistance to aminoglycoside antibiotics in the *ramA* mutant was expected due to the presence of the *aph* cassette used to inactivate *ramA*. Among 98 metabolite analogs and antibiotics to which the *ramA* mutant showed increased sensitivity/susceptibility, with 78 were antimicrobials and toxic compounds (Table S2). The PM assay of the *ramA* mutant verified susceptibility to compounds in the classes of β-lactam, organic solvent, phenicol, and tetracycline as previously reported [20], [25]. Increased susceptibility to biocides was also observed. Furthermore, the strain became hypersensitive to DNA intercalating agents including acriflavine (−3.6-fold), 9-Aminoacridine (−3.9-fold), 2-Phenylphenol (−7.8-fold), and proflavin (−4.0-fold) after *ramA* disruption. Interestingly, the mutant exhibited better metabolism of 10 carbon and 6 phosphorus sources (Table 2).

**Table 2:** Changes in carbon, and phosphate source utilization greater than or equal to 1.5-fold due to *ramA* inactivation comparing *S*. Typhimurium LTL*ramA::aph* to its parental strain *S*. Typhimurium LTL.

Mode of action	Compound	Difference in fold (A.U.)^a^
C-source	N-Acetyl-Neuraminic Acid	1.8 (18447)
	D-Fructose	2.0 (19846)
	L-Fucose	2.4 (23929)
	D-Galactose	2.5 (24681)
	α-D-Glucose	2.1 (21423)
	N-Acetyl-D-Glucosamine	2.0 (20490)
	D-Mannose	1.8 (17994)
	D-Mannitol	1.7 (16768)
	L-Rhamnose	1.8 (18377)
	Fumaric Acid	1.9 (19730)
P-source	Adenosine 3′-Monophosphate	3.1 (30703)
	Adenosine 2′,3′-Cyclic Monophosphate	3.2 (32083)
	Guanosine 2′,3′-Cyclic Monophosphate	2.4 (24537)
	Thymidine 3′-Monophosphate	2.8 (27588)
	Uridine 3′-Monophosphate	2.0 (19659)
	Uridine 5′-Monophosphate	1.5 (15399)

a fold equals to arbitrary unit (A.U.)/10,000.

**Figure 2:**
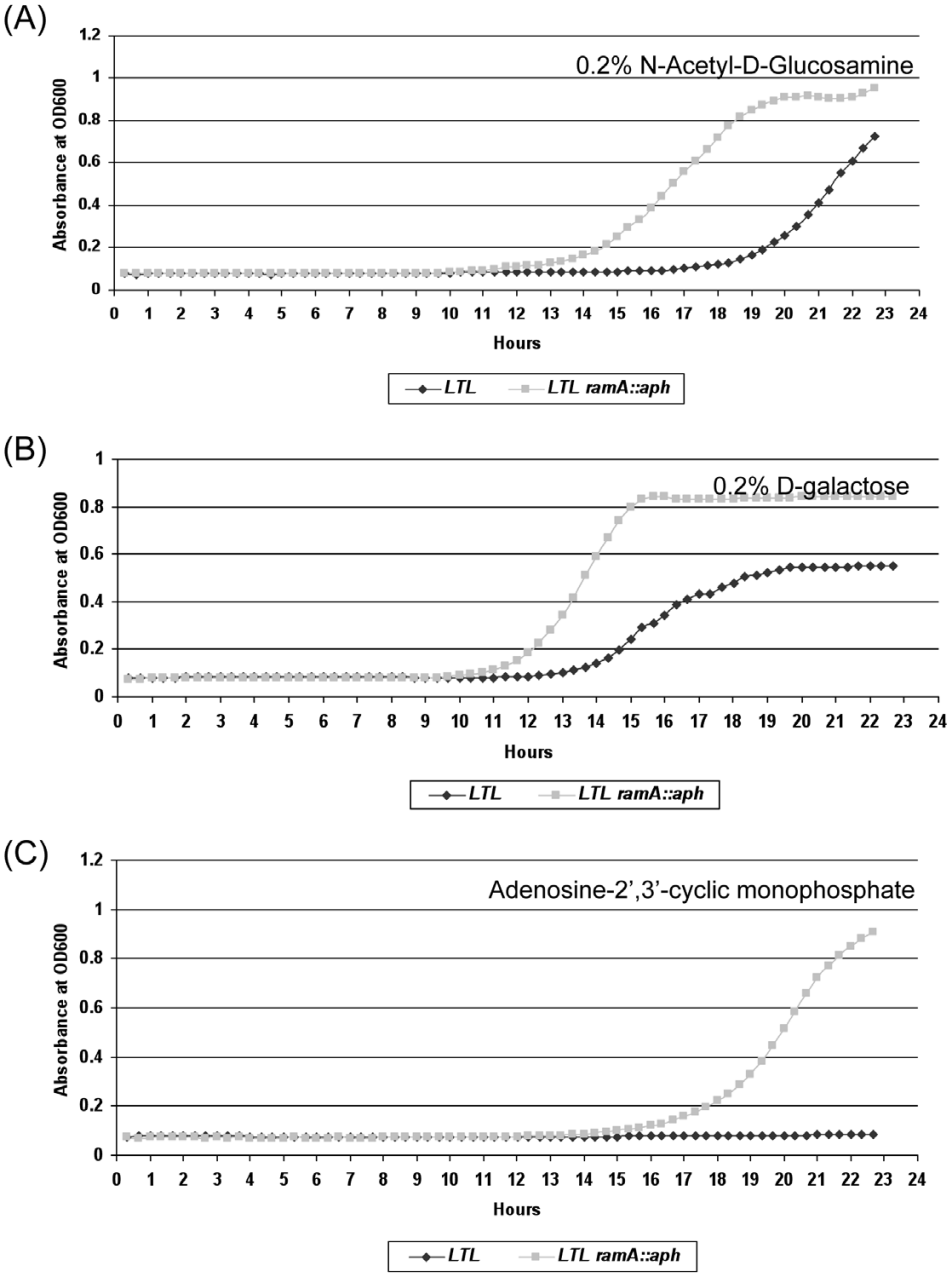
Growth kinetics of *S*. Typhimurium LTL and its *ramA* mutant grown in M9 minimal medium containing1×M9 salts, 2 mM MgSO_4_, and 0.1 mM CaCl_2_ with 0.2% N-Acetyl-D-Glucosamine, 0.2% D-galactose, and Adenosine-2′,3′-cyclic monophosphate, as carbon source or phosphate source, respectively. Growth curves were performed with a Bioscreen C Microbiology Reader from Labsystems. Cell growth was monitored at 37°C with shaking. Absorbance at OD 600 nm (A_600_) was measured and recorded every 20 min for 24 hrs.

Several findings in the PM analysis were subsequently confirmed by additional assays (Fig. 2 and Table 3). For example, in growth studies, parental strain LTL was defective (P<0.5) in using 0.2% N-Acetyl-D-glucosamine or 0.2% D-galactose as a carbon source, and 20 mM adenosine 2′, 3′ cyclic monophosphate (cAMP) as a phosphorus source (Fig. 2). By determining MICs using agar dilution, the *ramA* mutant showed increased susceptibility to promethazine (160 ug/ml), a cyclic nucleotide phosphodiesterase inhibitor [49], propranolol (240 ug/ml), a non-selective beta-adrenergic blocker, and acriflavine (16 µg/ml), a DNA intercalating agent.

**Table 3:** Antimicrobial activities of acriflavin, promethazine, and propranolol against *S*. Typhimurium strain LTL and its *ramA* mutant.

Antimicrobial Agent	MIC (µg/ml)	
	LTL	LTL*ramA::aph*
Acriflavin	>64	16
Promethazine	640	160
Propranolol	>480	240

## Discussion

The role of RamA as a transcriptional regulator has been associated with MDR in *Salmonella* and other *Enterobacteriaceae* [9], [11], [25], [50]. The present study sought to explore the role of *ramA* using total genome transcription analysis and phenotypic array analysis as a global regulator. The transcriptomic experiments in this study revealed that in response to the *ramA* inactivation there were considerable changes in gene expression. These included changes in genes related to MDR, genes co-regulated by other regulators such as *marA*, and *soxS*, as well as genes involved in the metabolic pathways. Such changes were also reflected in phenotype microarray analysis. The inactivation of *ramA* caused changes in the response of *Salmonella* to at least 100 compounds.

We previously confirmed that both AcrAB-TolC efflux pump activity and the S83F substitution in *gyrA* contributed to resistance to nalidixic acid and fluoroquinolones in *S*. Typhimurium strain LTL (unpublished data). The increased expression of *acrB* was associated with the activation of *ramA*. In the present study, decreased expression of *acrA*, *acrB*, *tolC*, and *micF*, and increased expression of ompF were observed in LTL*ramA::aph*. Our data clearly showed that *acrAB-tolC, ompF* and *micF* were regulated by *ramA* at the transcriptional level. No change in the expression of *marA*, *soxS*, and *rob* indicated lack of the involvement of these regulators in the development of MDR. It is likely that *ramA* activates the MDR cascade independently of *marA*. Consistent with results from a study by Ricci et al [17], our phenotypic microarray data demonstrated that the inactivation of *ramA* increased *Salmonella*'s susceptibility to an array of antimicrobials, confirming an important role of *ramA* in conferring MDR. Among those compounds, many are known or recently identified substrates of AcrAB efflux [18], [25].

Bailey et al. [26] recently reported that inactivation of *ramA* led to altered expression of 223 genes in *S.* Typhimurium SL1344, including increased expression of 14 SPI-1 genes and decreased expression of 3 SPI-2 genes. Although none of genes involved in pathogenicity was eligible to be listed in Table 1 (ratio less than 2-fold), real-time PCR showed genes including *fliA*, *prgK,* and *sipB* (Fig. 1) had over 1.5-fold enrichment when compared to strain LTL. Moreover, logFC value used to determine genes with significantly altered expression was much higher (at least 2 fold) than B value (log odds value) that was used in the Bailey's study [26]. If the same criteria were used, the disruption of *ramA* would result in significantly increased expression of at least 9 SPI-1 genes encoding *prgHIJK* and *sipABCDF* in this study. Similarly, both studies showed the disruption of *ramA* was associated with changes in expression of genes involved in virulence (such as those in SPI-1). However, the expression of genes responsible for amino acid biosynthetic pathways [26] except *his* operon was not significantly affected. Nevertheless, these results shed light to the complex of regulation network under *ramA*.

It is interesting to note that at least 10 of the differentially expressed genes in this study were regulated by MarA and SoxS in *E. coli* as well [22], [38], [39] (Table 1). Additionally, this study showed an overlapping regulation between *soxS* and *ramA* in *Salmonella* (Table 1); *nifJ*, encoding oxidoreductase that shuttles electrons from pyruvate to reduce nitrogenase [47]. In line with data from other studies [21], it is likely *ramA* also plays a role as a transcriptional regulator of antioxidant defense in *Salmonella*. Previous studies showed decreased expression of *rob* in *S*. Enteritidis was likely due to down-regulation by *soxS* and *marA* [51], [52]. We also found a putative transcriptional regulator (LysR family) was down-regulated due to the inactivation of *ramA*. Clearly, there is a cross talk between *ramA* and other global regulators. One caveat of this transcriptomic study was that the *ramA* knockout mutant was compared to its parent strain with GyrA S83F mutation rather than to a strain with a clean genetic background. However, based on previous studies [53], [54], S83 mutation in GyrA alone has no or least influence on global supercoiling. Our data also showed no statistically significant difference in both doubling time between LT2-wt (31.2±2.3 min, n = 6 generations) and LTL (32.0±3.4 min, n = 6 generations), and biofilm formation between LT2-wt (OD_550_ = 0.120±0.005) and LTL (OD_550_ = 0.123±0.008) [55], suggesting little impact on supercoiling by the S83 GyrA mutation. We felt confident that the genetic background of the experimental strains could not have introduced significant confounding factors for data interpretations.

The inactivation of *ramA* affected the response of *Salmonella* to many different chemicals. Of these compounds, acriflavine, β-lactams, chloramphenicols, fusidic acid, macrolides, novobiocin, puromycin, sulfonamides, tetracyclines and trimethoprim were known substrates of AcrAB-TolC system [1], [56], [57], [58]. Recently, Zhou et al. [36] and Bailey et al. [25] substantially expanded the range of compounds as substrates of AcrAB-TolC system using phenotype microarray. In this study, PM1, instead of PM11A, through PM20 were used to identify phenotypic difference exhibited by *ramA* mutant. The disruption of *ramA* conferred the susceptibility to a wide range of compounds but also led to a better utilization of 10 carbohydrate carbon sources and 6 phosphorus sources. Additionally, the carbon and phosphorus metabolic differences due to the inactivation of *ramA* occurred only after 24-h growth in these arrays. We sought to resolve the altered phenotypes by comparing to the microarray data. It is worthy to note that the transcription level of *galP*, which is in MFS family and responsible for galactose transportation had increased by 0.6-fold (B value: 0.5>0), and that the transcription level of *nagE*, which partly comprises the PTS system N-acetyl glucosamine specific transporter subunit II and is responsible for N-acetyl glucosamine transportation, had increased by 0.9-fold (B-value:6.3>0) in the *ramA* mutant. Both array findings may provide possible genetic basis for the altered phenotypes.

In summary, the data generated by the transcriptomic and phenotypic arrays suggested that *ramA* be a global transcriptional regulator that controls a set of genes with diverse physiological functions. It is possible that RamA plays a role in virulence regulation as well. Further studies on cross talk between global regulators including *marA*, *soxRS* and *ramA,* and the genes under their control in *Salmonella* may provide important details related to the mechanisms that govern how *Salmonella* enhance their fitness in new and challenging environmental landscapes.

## Supporting Information

Table S1 Real Time-PCR Primers used in validation of microarray data.
(DOC)

Table S2 Compounds with increased susceptibilities greater than or equal to 1.5-fold due to *ramA* inactivation in comparing *S*. Typhimurium LTL*ramA::aph* to its parental strain *S*. Typhimurium LTL.
(DOC)

## References

[1] Baucheron S, Tyler S, Boyd D, Mulvey M, Chaslus-Dancla E (2004). AcrAB-TolC directs efflux-mediated multidrug resistance in *Salmonella enterica* serovar typhimurium DT104.. Antimicrob Agents Chemother.

[2] Buckley A, Webber M, Cooles S, Randall L, La Ragione R (2006). The AcrAB-TolC efflux system of *Salmonella enterica* serovar Typhimurium plays a role in pathogenesis.. Cell Microbiol.

[3] Webber M, Bailey A, Blair J, Morgan E, Stevens M (2009). The global consequence of disruption of the AcrAB-TolC efflux pump in *Salmonella enterica* includes reduced expression of SPI-1 and other attributes required to infect the host.. J Bacteriol.

[4] Hachler H, Cohen S, Levy S (1991). marA, a regulated locus which controls expression of chromosomal multiple antibiotic resistance in *Escherichia coli*.. J Bacteriol.

[5] White D, Goldman J, Demple B, Levy S (1997). Role of the *acrAB* locus in organic solvent tolerance mediated by expression of *marA*, *soxS*, or *robA* in *Escherichia coli*.. J Bacteriol.

[6] Piddock L (2006). Clinically relevant chromosomally encoded multidrug resistance efflux pumps in bacteria.. Clin Microbiol Rev.

[7] O'Regan E, Quinn T, Pages J, McCusker M, Piddock L (2009). Multiple regulatory pathways associated with high-level ciprofloxacin and multidrug resistance in *Salmonella enterica* serovar enteritidis: involvement of RamA and other global regulators.. Antimicrob Agents Chemother.

[8] Gallegos M, Schleif R, Bairoch A, Hofmann K, Ramos J (1997). Arac/XylS family of transcriptional regulators.. Microbiol Mol Biol Rev.

[9] Chollet R, Chevalier J, Bollet C, Pages J, Davin-Regli A (2004). RamA is an alternate activator of the multidrug resistance cascade in *Enterobacter aerogenes*.. Antimicrob Agents Chemother.

[10] Keeney D, Ruzin A, Bradford P (2007). RamA, a transcriptional regulator, and AcrAB, an RND-type efflux pump, are associated with decreased susceptibility to tigecycline in *Enterobacter cloacae*.. Microb Drug Resist.

[11] van der Straaten T, Janssen R, Mevius D, van Dissel J (2004). Salmonella gene *rma* (*ramA*) and multiple-drug-resistant *Salmonella enterica* serovar typhimurium.. Antimicrob Agents Chemother.

[12] Yassien M, Ewis H, Lu C, Abdelal A (2002). Molecular cloning and characterization of the *Salmonella enterica* Serovar Paratyphi B *rma* Gene, which confers multiple drug resistance in *Escherichia coli*.. Antimicrob Agents Chemother.

[13] Schneiders T, Amyes S, Levy S (2003). Role of AcrR and RamA in fluoroquinolone resistance in clinical *Klebsiella pneumoniae* isolates from Singapore.. Antimicrob Agents Chemother.

[14] Ruzin A, Immermann F, Bradford P (2008). Real-time PCR and statistical analyses of *acrAB* and *ramA* expression in clinical isolates of *Klebsiella pneumoniae*.. Antimicrobial Agents And Chemotherapy.

[15] Feuerriegel S, Heisig P (2008). Role of Global Regulator Rma for Multidrug Efflux-Mediated Fluoroquinolone Resistance in *Salmonella*.. Microbial Drug Resistance.

[16] George A, Hall RM, Stokes H (1995). Multidrug resistance in *Klebsiella pneumoniae*: a novel gene, ramA, confers a multidrug resistance phenotype in Escherichia coli.. Microbiology.

[17] Ricci V, Piddock L (2009). Only for substrate antibiotics are a functional AcrAB-TolC efflux pump and RamA required to select multidrug-resistant *Salmonella* Typhimurium.. J Antimicrob Chemother.

[18] Abouzeed Y, Baucheron S, Cloeckaert A (2008). ramR mutations involved in efflux-mediated multidrug resistance in *Salmonella enterica* serovar Typhimurium.. Antimicrob Agents Chemother.

[19] Ricci V, Piddock L (2009). Ciprofloxacin selects for multidrug resistance in *Salmonella enterica* serovar Typhimurium mediated by at least two different pathways.. J Antimicrob Chemother.

[20] Zheng J, Cui S, Meng J (2009). Effect of transcriptional activators RamA and SoxS on expression of multidrug efflux pumps AcrAB and AcrEF in fluoroquinolone-resistant *Salmonella* Typhimurium.. J Antimicrob Chemother.

[21] van der Straaten T, Zulianello L, van Diepen A, Granger D, Janssen R (2004). *Salmonella enterica* serovar Typhimurium RamA, intracellular oxidative stress response, and bacterial virulence.. Infect Immun.

[22] Barbosa T, Levy S (2000). Differential expression of over 60 chromosomal genes in *Escherichia coli* by constitutive expression of MarA.. J Bacteriol.

[23] Miller P, Sulavik M (1996). Overlaps and parallels in the regulation of intrinsic multiple-antibiotic resistance in *Escherichia coli*.. Mol Microbiol.

[24] Nikaido E, Yamaguchi A, Nishino K (2008). AcrAB multidrug efflux pump regulation in *Salmonella enterica* serovar Typhimurium by RamA in response to environmental signals.. J Biol Chem.

[25] Bailey AM, Paulsen IT, Piddock LJ (2008). RamA confers multidrug resistance in *Salmonella enterica* via increased expression of *acrB*, which is inhibited by chlorpromazine.. Antimicrob Agents Chemother.

[26] Bailey AM, Ivens A, Kingsley R, Cottell JL, Wain J (2010). RamA, a member of the AraC/XylS family, influences both virulence and efflux in *Salmonella enterica* serovar Typhimurium.. Journal of Bacteriology.

[27] Datsenko K, Wanner B (2000). One-step inactivation of chromosomal genes in *Escherichia coli* K-12 using PCR products.. Proc Natl Acad Sci U S A.

[28] Bearson B, Bearson S (2008). The role of the QseC quorum-sensing sensor kinase in colonization and norepinephrine-enhanced motility of *Salmonella enterica* serovar Typhimurium.. Microb Pathog.

[29] Smyth G (2004). Linear models and empirical bayes methods for assessing differential expression in microarray experiments.. Stat Appl Genet Mol Biol.

[30] Yang Y, Dudoit S, Luu P, Lin D, Peng V (2002). Normalization for cDNA microarray data: a robust composite method addressing single and multiple slide systematic variation.. Nucleic Acids Res.

[31] Bolstad B, Irizarry R, Astrand M, Speed T (2003). A comparison of normalization methods for high density oligonucleotide array data based on variance and bias.. Bioinformatics.

[32] Benjamini Y, Hochberg Y (1995). Controlling the false discovery rate: a practical and powerful approach to multiple testing.. J Roy Statist Soc Ser B.

[33] Miron M, Woody O, Marcil A, Murie C, Sladek R (2006). A methodology for global validation of microarray experiments.. BMC Bioinformatics.

[34] Botteldoorn N, Van Coillie E, Grijspeerdt K, Werbrouck H, Haesebrouck F (2006). Real-time reverse transcription PCR for the quantification of the *mntH* expression of *Salmonella enterica* as a function of growth phase and phagosome-like conditions.. J Microbiol Methods.

[35] Bochner B, Gadzinski P, Panomitros E (2001). Phenotype microarrays for high-throughput phenotypic testing and assay of gene function.. Genome Res.

[36] Zhou L, Lei X, Bochner B, Wanner B (2003). Phenotype microarray analysis of *Escherichia coli* K-12 mutants with deletions of all two-component systems.. J Bacteriol.

[37] CLSI/NCCLS, Clinical and Laboratory Standards Institution/NCCLS, Wayne PA (2002). Performance standards for antimicrobial disk and dilution susceptibility tests for bacteria isolated from animals.. Approved Standard-Second Edition.CLSI/NCCLS document M31-A2.

[38] Hidalgo E, Ding H, Demple B (1997). Redox signal transduction: mutations shifting [2Fe-2S] centers of the SoxR sensor-regulator to the oxidized form.. Cell.

[39] Liochev SI, Hausladen A, Fridovich I (1999). Nitroreductase A is regulated as a member of the *soxRS* regulon of *Escherichia coli*.. Proc Natl Acad Sci U S A.

[40] Ariza R, Cohen S, Bachhawat N, Levy S, Demple B (1994). Repressor mutations in the *marRAB* operon that activate oxidative stress genes and multiple antibiotic resistance in *Escherichia coli*.. J Bacteriol.

[41] Greenberg J, Chou J, Monach P, Demple B (1991). Activation of oxidative stress genes by mutations at the *soxQ/cfxB/marA* locus of *Escherichia coli*.. J Bacteriol.

[42] Nishino K, Latifi T, Groisman EA (2006). Virulence and drug resistance roles of multidrug efflux systems of *Salmonella enterica* serovar Typhimurium.. Molecular Microbiology.

[43] Nishino K, Yamada J, Hirakawa H, Hirata T, Yamaguchi A (2003). Roles of TolC-dependent multidrug transporters of *Escherichia coli* in resistance to beta-lactams.. Antimicrob Agents Chemother.

[44] Cohen S, McMurry L, Levy S (1988). marA locus causes decreased expression of OmpF porin in multiple-antibiotic-resistant (Mar) mutants of *Escherichia coli*.. J Bacteriol.

[45] Delihas N (1997). Antisense *micF* RNA and 5′-UTR of the target *ompF* RNA: phylogenetic conservation of primary and secondary structures..

[46] Olliver A, Valle M, Chaslus-Dancla E, Cloeckaert A (2004). Role of an *acrR* mutation in multidrug resistance of in vitro-selected fluoroquinolone-resistant mutants of *Salmonella enterica* serovar Typhimurium.. FEMS Microbiol Lett.

[47] Pomposiello PJ, Demple B (2000). Identification of SoxS-regulated genes in *Salmonella enterica* serovar typhimurium.. J Bacteriol.

[48] Spory A, Bosserhoff A, von Rhein C, Goebel W, Ludwig A (2002). Differential regulation of multiple proteins of *Escherichia coli* and *Salmonella enterica* serovar Typhimurium by the transcriptional regulator SlyA.. J Bacteriol.

[49] Levin RM, Weiss B (1976). Mechanism by which psychotropic drugs inhibit adenosine cyclic 3′,5′-monophosphate phosphodiesterase of brain.. Mol Pharmacol.

[50] Komatsu T, Ohta M, Kido N, Arakawa Y, Ito H (1990). Molecular characterization of an *Enterobacter cloacae* gene (romA) which pleiotropically inhibits the expression of *Escherichia coli* outer membrane proteins.. J Bacteriol.

[51] Schneiders T, Levy SB (2006). MarA-mediated transcriptional repression of the *rob* promoter.. J Biol Chem.

[52] Michan C, Manchado M, Pueyo C (2002). SoxRS down-regulation of *rob* transcription.. J Bacteriol.

[53] Bagel S, Hullen V, Wiedemann B, Heisig P (1999). Impact of *gyrA* and *parC* mutations on quinolone resistance, doubling time, and supercoiling degree of *Escherichia coli*.. Antimicrob Agents Chemother.

[54] Aleixandre V, Urios A, Herrera G, Blanco M (1989). New *Escherichia coli gyrA* and *gyrB* mutations which have a graded effect on DNA supercoiling.. Mol Gen Genet.

[55] O'Toole GA, Kolter R (1998). Flagellar and twitching motility are necessary for *Pseudomonas aeruginosa* biofilm development.. Mol Microbiol.

[56] Ma D, Alberti M, Lynch C, Nikaido H, Hearst JE (1996). The local repressor AcrR plays a modulating role in the regulation of *acrAB* genes of *Escherichia coli* by global stress signals.. Mol Microbiol.

[57] Ma D, Cook DN, Alberti M, Pon NG, Nikaido H (1993). Molecular cloning and characterization of *acrA* and *acrE* genes of *Escherichia coli*.. J Bacteriol.

[58] Sulavik MC, Houseweart C, Cramer C, Jiwani N, Murgolo N (2001). Antibiotic susceptibility profiles of *Escherichia coli* strains lacking multidrug efflux pump genes.. Antimicrob Agents Chemother.

