# Supplementary material for: Differential Gene Expression by RamA in Ciprofloxacin-Resistant Salmonella Typhimurium
Source: PLoS One. 2011 Jul 19;6(7):e22161. doi: 10.1371/journal.pone.0022161 (PMC3139621; doi:10.1371/journal.pone.0022161)
Supplement: Table S1 — Real Time-PCR Primers used in validation of microarray data. (DOC) [file pone.0022161.s001.doc]

Table S1. Real Time-PCR Primers used in validation of microarray data

| Locus^a^ | Gene | Primer sequence^c^ | Size (bp) |
| --- | --- | --- | --- |
|
| STM0476 | *acrA* | 5’-CCCGGATCACACCTTATTGC-3’  5’-CTGGCTTGCGACGATTTG-3’ | 178 |
| STM2481 | *acrD* | 5’-ACGGTTCCGAGGTCAAACTG-3’  5’-TCGCGGTCGCCATTTC-3’ | 144 |
| STM0477 | *acrR* | 5’-CAAGGCGTATCGGCAACC-3’  5’-TAAGCGGCGTCGTTCTTCTG-3’ | 246 |
| STM1916 | *cheY* | 5’-TTATCTCCGACTGGAACATGC-3’  5’-GACCATCAACACGGGTAACG-3’ | 98 |
| STM0260 | *dniR* | 5’-GCTGGCTTCCGGTGAGATTG-3’  5’-CCACGCAGGTTGTTCCACTG-3’ | 165 |
| STM3385 | *fis* | 5’-CCTGCGTGACTCGGTTAAAC-3’  5’-ATTGCATCACCATGTCCAAC-3’ | 128 |
| STM1956 | *fliA* | 5’-TGGATAAACACTCGCTGTGG-3’  5’-GGTAAATGCCGTTCCTTGC-3’ | 173 |
| STM1660.S | *fnr* | 5’-GAATTCCGCCTGACGATGAC-3’  5’-ACCTTTCACCGCCAGCATAC-3’ | 117 |
| STM0087 | *folA* | 5’-CCGGAAATTATGGTCATTGG-3’  5’-CGTCGGCATCGTGAAAC-3’ | 169 |
| STM4084 | *fpr* | 5’-ACGGTGCCGCAAGGAAAGC-3’  5’-CGGTCGCCAGCATCCATAGC-3’ | 139 |
| STM0473 | *hha* | 5’-TGATTATTTGATGCGTTTAC-3’  5’-GGATCTTGTCGTAGAGTTTG-3’ | 164 |
| STM4237 | *lexA* | 5’-TCGCGAGGTATCCGTCTG-3’  5’-TATGTACCGCCAGCAAATCC-3’ | 217 |
| STM0866 | *mdfA* | 5’-TTGCCGGTAATCTGGTGTTG-3’  5’-GCTTAATCCCGCCGTCATC-3’ | 158 |
| STM1651 | *nifJ* | 5’-GCGACCATTCCGATGTGATG-3’  5’-TTCAGCGTAGCCCTGTGTGC-3’ | 112 |
| STM0999 | *ompF* | 5’-CGTGCTGGCGGTTTGTTGAC-3’  5’-TTGCTGTACGCTGCGGTGAC-3’ | 188 |
| STM1230 | *phoQ* | 5’-TCAGCGCGCCGATACCCTAC-3’  5’-GCGCCACCGAGCAGACTGTC-3’ | 114 |
| STM2871 | *prgK* | 5’-TGGAAATAGCGCAGATGTTC-3’  5’-GGACCCTGGCGGAGAG-3’ | 138 |
| STM2924 | *rpoS* | 5’-CGTTATGGCAATCGTGGACTG-3’  5’-CGAATCGTACGGGTTTGGTTC-3’ | 176 |
| STM2885 | *sipB* | 5’-ACGCGCAAAGCCGAGGAAAC-3’  5’-CCCGTCGCCGCCTTCAC-3’ | 176 |
| STM4055 | *sodA* | 5’-GCGATCTGAAAGCGGCTATC-3’  5’-GGAATCCTGGTTTGCGGTAG-3’ | 158 |
| STM3186 | *tolC* | 5’-GTGACCGCCCGCAACAAC-3’  5’-ATTCAGCGTCGGCAGGTGAC-3’ | 243 |
| STM0173 | *yadH* | 5’-TGTCGGCGGCGGCGTAG-3’  5’-GCAGTCCGGCCAGCGAGAAC-3’ | 149 |
| STM2268 | *micF* | 5’-TTGAAATAGGGGTAAACAGAC-3’  5’-TCTTCATTCGCAACTAAAATA-3’ | 93 |
| STM2659 | *rrsG* | 5’-GTTACCCGCAGAAGAAGCAC-3’  5’- CACATCCGACTTGACAGACC 3’ | 123 |

^a^. Location of the ORF in the serovar Typhimurium LT2 genome.
