# Supplementary material for: Differential Gene Expression by RamA in Ciprofloxacin-Resistant Salmonella Typhimurium
Source: PLoS One. 2011 Jul 19;6(7):e22161. doi: 10.1371/journal.pone.0022161 (PMC3139621; doi:10.1371/journal.pone.0022161)
Supplement: Table S2 — Compounds with increased susceptibilities greater than or equal to 1.5-fold due to ramA inactivation in comparing S. Typhimurium LTLramA::aph to its parental strain S. Typhimurium LTL. (DOC) [file pone.0022161.s002.doc]

Table S2. Compounds with increased susceptibilities greater than or equal to 1.5-fold due to *ramA* inactivation in comparing *S*. Typhimurium LTL*ramA::aph* to its parental strain *S*. Typhimurium LTL

| Mode of Action | Compound | Difference in fold (A.U.)^a^ |
| --- | --- | --- |
| ATPase, Na^+^/K^+^ and Mg^++^ | Sanguinarine | -2.1 (-21381) |
| beta-adrenergic blocker | Propranolol | -4.8 (-47602) |
| cell cycle modulation | Chlorpromazine | -7.1 (-70954) |
| cell wall, cephalosporin first generation | Cefazolin | -2.4 (-23551) |
|  | Cephalothin | -6.2 (-62162) |
| cell wall, cephalosporin second generation | Cefuroxime | -4.1 (-40644) |
|  | Cefoxitin | -4.6 (-46274) |
|  | Cefmetazole | -9.1 (-91249) |
|  | Cefamandole | -1.9 (-19821) |
| cell wall, cephalosporin third generation | Cefotaxime | -10.1 (-100895) |
| cell wall, lactam | Amoxicillin | -2.3 (-23333) |
|  | Cloxacillin | -2.5 (-24579) |
|  | Nafcillin | -2.1 (-20881) |
|  | Penicillin G | -6.8 (-67977) |
|  | Carbenicillin | -7.6 (-76353) |
|  | Oxacillin | -7.9 (-79824) |
|  | Ampicillin | -2.0 (-19931) |
|  | Azlocillin | -5.1 (-50750) |
|  | Moxalactam | -4.8 (-48232) |
|  | Carbenicillin | -9.6 (-96026) |
|  | Aztreonam | -2.8 (-28216) |
|  | Phenethicillin | -8.1 (-81121) |
| chelator, lipophilic | 2,2'-Dipyridyl | -1.5 (-15465) |
|  | 1,10-Phenanthroline | -3.9 (-38568) |
|  | Fusaric acid | -1.6 (-15933) |
|  | 5,7-Dichloro-8-hydroxyquinoline | -4.3 (-42795) |
|  | 5-Chloro-7-iodo-8-hydroxyquinoline | -2.1 (-21370) |
| cholinergic antagonist | Orphenadrine | -4.3 (-43365) |
|  | Pridinol | -3.0 (-30048) |
| cyclic nucleotide phosphodiesterase | Promethazine | -6.6 (-66479) |
| DNA damage, nitrofuran analog | Furaltadone | -2.2 (-21628) |
| DNA intercalator | Acriflavine | -3.6 (-36308) |
|  | 9-Aminoacridine | -3.9 (-38602) |
|  | 2-Phenylphenol | -7.8 (-77595) |
|  | Proflavine | -4.0 (-40050) |
| DNA unwinding, gyrase, topoisomerase | Novobiocin | -1.7 (-17148) |
| folate antagonist | Sulfamonomethoxine | -6.6 (-66444) |
| folate synthesis | Trimethoprim | -7.1 (-70899) |
|  | Sulfachloropyridazine | -1.9 (-19358) |
|  | Sulfisoxazole | -2.1 (-20690) |
| fungicide | Chloroxylenol | -6.8 (-67905) |
| imidazoline binding sites, agonist | Harmane | -1.7 (-16973) |
| lipid synthesis, fatty acid inhibitor | Triclosan | -1.8 (-17723) |
| membrane permeability, guanidine, fungicide | Dodine | -4.8 (-48464) |
| membrane, detergent, cationic | Dodecyltrimethyl ammonium bromide | -2.8 (-28444) |
|  | Cetylpyridinium chloride | -1.6 (-15664) |
|  | Methyltrioctylammonium chloride | -3.6 (-35461) |
|  | Domiphen bromide | -5.0 (-50426) |
| membrane, disorganize structure | Polymyxin B | -1.6 (-15555) |
| membrane, electron transport | Chlorhexidine | -4.3 (-43557) |
| membrane, transport | Amitriptyline | -6.1 (-60765) |
| N-Source,amino acid | L-Cysteine | -1.6 (-16316) |
| pH, deaminase | pH 9.5 + L-Tryptophan | -1.6 (-15874) |
|  | pH 9.5 + Histamine | -2.4 (-24260) |
|  | pH 9.5 + Phenylethylamine | -2.1 (-20608) |
| protein kinase C inhibitor | Chelerythrine | -2.3 (-22862) |
| protein synthesis | Chloramphenicol | -7.3 (-72909) |
| protein synthesis, 30S ribosomal subunit | Puromycin | -4.5 (-45334) |
| protein synthesis, 30S ribosomal subunit, tetracycline | Chlortetracycline | -4.2 (-41652) |
|  | Minocycline | -11.1(-111368) |
|  | Demeclocyline | -6.1 (-60722) |
|  | Tetracycline | -1.8 (-17902) |
|  | Penimepicycline | -6.7 (-66963) |
|  | Rolitetracycline | -6.5 (-64853) |
|  | Oxytetracycline | -4.5 (-44811) |
|  | Doxycycline | -7.5 (-75171) |
| protein synthesis, 50S ribosomal subunit, 23S rRNA | Thiamphenicol | -7.5 (-75086) |
| protein synthesis, 50S ribosomal subunit, macrolide | Spiramycin | -1.8 (-18272) |
|  | Josamycin | -1.9 (-19462) |
| protein synthesis, elongation factor | Fusidic acid | -1.5 (-15272) |
| protein synthesis, lincosamide | Lincomycin | -2.8 (-27691) |
| respiration | Iodonitro tetrazolium violet | -1.8 (-17902) |
|  | 18-Crown-6 ether | -2.4 (-24234) |
|  | Thioridazine | -4.4 (-44295) |
| toxic anion | Potassium tellurite | -1.9 (-19157) |
|  | Cadmium chloride | -2.2 (-22386) |
|  | Lithium chloride | -1.6 (-15959) |
| tRNA synthetase | Glycine hydroxamate | -4.4 (-44588) |

^a^ fold equals to arbitrary unit (A.U.)/10,000
